# Supplementary material for: Using a design-based research approach to develop a technology-supported physical education course to increase the physical activity levels of university students: Study protocol paper
Source: PLoS One. 2022 Dec 1;17(12):e0269759. doi: 10.1371/journal.pone.0269759 (PMC9714829; doi:10.1371/journal.pone.0269759)
Supplement: S1 Appendix — (DOCX) [file pone.0269759.s001.docx]

**S1 Appendix. Semi-structured Focus Group Discussion Guidelines**

# Semi-structured Focus Group Discussion Guideline (Key Stakeholders) – Phase 1

**Participants -** a physical education course lecturer**,** a physical education course coordinator, a member of the curriculum development team, and the Dean of the Faculty of Sport and Health Education) at Universitas Pendidikan Indonesia.

**Summary of this Instrument -** A semi-structured group discussion focussing on key issues from the key stakeholders.

**Research instrument and protocol -**

The purpose of the focus group is to brainstorm ideas as to how to design the proposed technology-based physical education course to increase the physical activity levels of university students enrolled in the course. Three key stakeholders will be invited to attend a meeting to discuss issues related to development of a technology-based physical education course. The researcher will chair the session. A brief introduction to the research, demographic questionnaire and participation information statements will be provided. The session will continue to those who consent to being a participant.

**Audio recorded** – 60 minutes

**Agenda for focus group discussion –** Completion of demographic data (5 mins); Introduction and overview (5 mins); Semi-structured group discussion (50 mins)

**Demographic data**

*Please complete and return the demographic questionnaire before the focus group discussions begin!*

**Introduction and overview**

Thank you so much for participating in this research project and focus group discussions.

This research project is being conducted by myself as a part of my PhD project, supervised by A/Prof Wayne Cotton, PhD and Dr Louisa Peralta, PhD at The University of Sydney, Australia. The aim of this research is to create a series of design principles that inform the development of a technology-based physical education course that improves the physical activity levels of university students. You have been invited to participate in this study because you are key stakeholders and practitioners of the physical education course (you are a physical education course coordinator, part of the curriculum development team, or the Dean of the Faculty of Sport and Health Education) at Universitas Pendidikan Indonesia.

In this focus group discussion, we are interested in your experiences. Please respond to the questions as honestly as you can, as there are no right or wrong answers and no trick questions. We simply want to know more about the physical education course at Universitas Pendidikan Indonesia. Your responses will be held in confidence and only used for our research purposes.

**Do you give your consent to be audio-recorded during this focus group discussion?**

1. What do you know about the general physical education course at Universitas Pendidikan Indonesia?
   1. Prompt: Is it required or an elective course for all student across faculty?
   2. Have you taught the physical education course?
   3. How long you taught the course?
2. Can you tell me who are the lecturers or course instructor for physical education course?
3. Can you tell me how the lecturer or course instructor is selected to teach the course? Are there any specific requirements for the lecturer or course instructor? Is there any teacher training or meetings with the teacher or course instructor before the course starts?
4. Can you tell me about the syllabi and modules of the physical education course?
5. Are there any specific/standardized syllabi and modules?
6. Who developed the syllabi and modules?
7. Can you tell me how the university to monitors syllabi or modules implementation?
8. Can you tell me about the main outcome/s of the general physical education course at Universitas Pendidikan Indonesia?
9. Prompt: to what extent has a long-life physical activity been an outcome of the physical education course?
10. Do you think the students have equal opportunities to achieve this outcome?
11. How do you asses the student’s achievement of the outcomes?
12. Could you explain the strengths for managing and/or teaching the physical education course?
13. Could you explain the challenges for managing and/or teaching the physical education course? How have you tried to minimise or overcome these?
14. Can you tell me more about the current technologies used to support the physical education course?
15. What other technologies could help you to deliver the physical education course? Provide the rationale for these suggestions.
16. What do you think must be included in a technology-based physical education for increasing student’s physical activity levels? What are the key things that must be included? What features should be included?
17. What do you think if the physical education course provides physical activity knowledge with extensive feedback and interaction between teachers and students?
18. What do you think if the physical education course utilises a simple and familiar technological device?
19. What do you think if the physical education course allows students to create individual goal setting, track their achievement and for personalised feedback from the teacher?
20. What do you think if the physical education course provides exercise examples that guide students to do more physical activity in their own time, independently and outside of the dedicated course time?
21. Is there any additional comments?

# Semi-structured Focus Group Discussion Guideline (Lecturers) – Phase 1

**Participants -** Up to 6 general physical education lecturers who have experience in teaching the course.

**Summary of this Instrument -** A semi-structured group discussion focussing on key issues from the course instructors/teacher’s perspective.

**Research instrument and protocol -**

The purpose of the focus group is to brainstorm ideas as to how to design the proposed technology-based physical education course to increase the physical activity levels of university students enrolled in the course. A group of six teachers will be invited to attend a meeting to discuss issues related to development of a technology-based physical education course. The researcher will chair the session. A brief introduction to the research and participation information statements will be provided. The session will continue to those who consent to be participants.

**Audio recorded** – 60 minutes

**Agenda for focus group discussion –** Completing demographic data (5 mins); Introduction and overview (5 mins); Semi-structured group discussion (50 mins)

**Demographic data:**

*Please complete and return the demographic questionnaire before the focus group discussion begin!*

**Introduction and overview**

Thank you so much for participating in this research project and focus group.

This research project is being conducted by myself as a part of my PhD project, supervised by A/Prof Wayne Cotton, PhD and Dr Louisa Peralta, PhD at The University of Sydney, Australia. The aim of this research is to create a series of design principles that inform the development of a technology-based physical education course that improves the physical activity levels of university students. You have been invited to participate in this study because you are current or past teacher/course instructor of the physical education course at Universitas Pendidikan Indonesia.

In this focus group discussion, we are interested in your experiences as a physical education course lecturers/course instructor at Universitas Pendidikan Indonesia. Please respond to the questions as honestly as you can, as there are no right or wrong answers and no trick questions. We simply want to know more about the physical education course at Universitas Pendidikan Indonesia. Your responses will be held in confidence and only used for our research purposes.

**Semi-structured group discussion**

**Do you give your consent to be audio-recorded during this focus group discussion?**

1. Can you tell me how you are selected as lecturer or course instructor to teach the course? Are there any teacher training or meetings (formal or informal) with the course coordinator or teachers before the course starts?
2. Can you tell me about the syllabi and modules of the physical education course?
3. Prompt: Are there any specific/standardized syllabi and modules? If no, did you develop your own syllabi and modules?
4. How confident you use the syllabi and modules?
5. Can you tell me how the university to monitors syllabi or modules implementation?
6. Can you tell me about the main outcome/s of the general physical education course at Universitas Pendidikan Indonesia?
7. Prompt: to what extent has a long-life physical activity been an outcome of the physical education course?
8. Do you think the students have equal opportunities to achieve this outcome?
9. How do you assess the student’s achievement of the outcomes?
10. Could you explain the challenges for teaching the physical education course? How have you tried to minimise or overcome these?
11. Can you tell me more about the current technologies used to support the physical education course?
12. What other technologies do you think could help you to deliver the physical education course? Provide the rationale for these suggestions.
13. What do you think must be included in a technology-based physical education for increasing student’s physical activity levels? Prompt: What are the key points that must be included? What features should be included?
14. What do you think if the physical education course provides physical activity knowledge with extensive feedback and interaction between teachers and students?
15. What do you think if the physical education course utilises a simple and familiar technological device?
16. What do you think if the physical education course allows students to create individual goal setting, track their achievement and for personalised feedback from the teacher?
17. What do you think if the physical education course provides exercise examples that guide students to do more physical activity in their own time, independently and outside of the dedicated course time?
18. Do you have any additional comments?

# Semi-structured Focus Group Discussion Guideline (Students) – Phase 1

**Participants -** Up to 6 students who have enrolled in the physical education course at Universitas Pendidikan Indonesia.

**Summary of this Instrument -** A semi-structured group discussion of the key issues from the students’ perspective.

**Research instrument and protocol -**

The purpose of the focus group is to brainstorm ideas as to how to design the proposed technology-based physical education course for increasing the physical activity levels of university students. A group of six students who have completed the course will be invited to discuss their experiences of the course. The researcher will chair the session. A brief introduction to the research and participation information statements will be provided. The session will continue to those who consent to be participants.

**Audio recorded** – 60 minutes

**Agenda for focus group discussion –** Completing demographic data (5 mins); Introduction and overview (5 mins); Semi-structured group discussion (50 mins)

**Demographic data:**

*Please complete and return the demographic questionnaire before the focus group discussion begin!*

**Introduction and overview (5 mins)**

Thank you so much for participating in this research project and focus group.

This research project is being conducted by myself as a part of my PhD project, supervised by A/Prof Wayne Cotton, PhD and Dr Louisa Peralta, PhD at The University of Sydney, Australia. This research aims to create a series of design principles to inform the development of a technology-based physical education course that improves the physical activity levels of university students. You have been invited to participate in this study because you are students who have completed the general physical education course at Universitas Pendidikan Indonesia.

**Focus Group discussion (50 mins)**

In this focus group discussion, we are interested in your experiences in enrolling in the physical education course at Universitas Pendidikan Indonesia. Please respond to the questions as honestly as you can, as there are no right or wrong answers and no trick questions. We simply want to know more about the physical education course at Universitas Pendidikan Indonesia. Your responses will be held in confidence and only used for our research purposes.

**Do you give your consent to be audio-recorded during this focus group discussion?**

1. Have you completed the physical education course? Is it a required or elective course? If elective, why did you choose this course?
2. Can you tell me what did you learn from the course?
3. Can you tell me what did you like the most from the course? What did you not like about the course? What suggestions do you have to improve the course?
4. Do you believe that you are more active after completed the course? Why?
5. Can you tell me, what technology was used in the course that helped to support your learning in the physical education course?
6. What new technology do you think could be included in the course to assist a students learning and increase their physical activity levels? What are the key features that must be included?
7. Do you have any additional comments?
