# Supplementary material for: Using a design-based research approach to develop a technology-supported physical education course to increase the physical activity levels of university students: Study protocol paper
Source: PLoS One. 2022 Dec 1;17(12):e0269759. doi: 10.1371/journal.pone.0269759 (PMC9714829; doi:10.1371/journal.pone.0269759)
Supplement: S1 File — (PDF) [file pone.0269759.s003.pdf]

A Technology-Supported Physical Education Course to Increase the Physical Activity Levels of University Students: A Randomised Controlled Trial

A TECHNOLOGY-SUPPORTED PE COURSE TO INCREASE THE PA LEVELS OF UNIVERSITY STUDENTS: RCT

STATEMENT OF COMPLIANCE FOR NON DRUG OR DEVICE CLINICAL TRIALS

This document is a protocol for a clinical research study. The study will be conducted in compliance with all stipulations of this protocol, the conditions of ethics committee approval, the NHMRC National Statement on Ethical Conduct in Human Research (as updated) and the Handbook for Good Clinical Research Practice (GCP). The Therapeutic Goods Act has adopted ICH Guideline for Good Clinical Practice.

Contents

STATEMENT OF COMPLIANCE FOR NON DRUG OR DEVICE CLINICAL TRIALS **2**

1. GENERAL INFORMATION **4**

2. SYNOPSIS **4**

3. RATIONALE / BACKGROUND **5**

4. AIMS / OBJECTIVES / HYPOTHESES **6**

5. PARTICIPATING SITES **6**

6. RESEARCH PLAN / STUDY DESIGN **7**

6.1 TYPE OF STUDY 7
6.2 SAMPLE SIZE INCLUDING POWER CALCULATION 7
6.3 STATISTICAL ANALYSES 7
6.4 RECRUITMENT AND SELECTION OF PARTICIPANTS* 8
6.5 INFORMED CONSENT PROCESS 8
6.6 INTERVENTION 9

7. ETHICAL CONSIDERATIONS **11**

8. SAFETY CONSIDERATIONS **12**

9. OUTCOMES **12**

9.1 PRIMARY OUTCOME 12
9.2 SECONDARY OUTCOME(S) 13

10. DATA MANAGEMENT **13**

11. TIMELINES / MILESTONES **13**

12. FINANCIAL **13**

13. PUBLICATION POLICY / DISSEMINATION OF RESULTS **14**

14. REFERENCES **14**

1. GENERAL INFORMATION

Title: A technology-based physical education course to increase university students physical activity levels: A Randomised controlled trial (RCT)

Chief Investigator: Dr Wayne Cotton

Primary Faculty/Department: School of Education and Social Work Research Operations; Faculty of Arts and Social Sciences

Investigators: Cotton Wayne; Peralta Louisa; Sultoni Kuston;

2. SYNOPSIS

**TITLE**	*A technology-supported physical education course to increase the physical activity levels of university students: A randomised controlled trial*
**OBJECTIVES**	*A prototype LMS, incorporated within a mobile phone app, was specifically designed and built to support a physical education course learning process at an Indonesian university. The app aimed to increase university students' physical activity levels, knowledge, and motivation. The prototype was tested and modified in 2021 with a small sample size. The randomised controlled trial (RCT) will recruit a larger sample size with the aim to test and evaluate the modified prototype Learning Management System (LMS) and mobile app. All students will receive the same content. Students in the intervention group will have access to the modified prototype mobile app that incorporates the LMS. This will enable them to access course content, feedback via their smartphone.*
**PRIMARY HYPOTHESIS**	*A technology-supported physical education course intervention will increase university student's physical activity levels, knowledge and motivation more effectively than non-technology-supported physical education courses.*
**DESIGN**	*This is a two-arm parallel, randomised controlled trial of a technology-supported physical education course intervention for students enrolled in an elective unit of study.*
**BLINDING/MASKING**	*The lecturers (n=6) will not be blinded as those randomly assigned to the intervention group (n=3) will be involved in professional development before they implement the intervention.*
**OUTCOMES**	*Primary outcome: Difference in students' physical activity levels, knowledge and motivation at post-test (at the completion of a 16-week semester) compared with the control group.*<br>*Secondary outcome: Student's experience and enjoyment of the technology-supported physical education course.*
**STUDY DURATION**	*16 weeks.*
**INTERVENTION/S**	*A 16-week physical education course incorporating the modified prototype Learning Management System (LMS) and mobile app.*
**NUMBER OF PARTICIPANTS**	*6 lecturers (a) n=3 will be assigned to the intervention group, (b) n=3 will be assigned to the control group.*<br>*n=300 students will be randomised 1:1 to either an intervention arm (a) n=150 a technology-supported physical education group or (b) n=150 usual physical education.*

**POPULATION**	*University students who enrol physical education course at Universitas Pendidikan Indonesia, Bandung, Indonesia*
**SELECTION AND ENROLMENT**	*Students will be included in the RCT if they meet the following inclusion criteria: 1) students enrolled in the elective physical education course with a lecturer who will participate in the RCT study; 2) voluntarily participate in the RCT study; and 3) owns an android smartphone.*

3. RATIONALE / BACKGROUND

Interventions focusing on increasing physical activity levels among various age groups from early childhood to the elderly have been growing over the last five years[1-4]. These studies have suggested that targeting physical activity at different time points across the lifespan is essential, especially when there is a transition related to educational events[5]. For example, the transition from preschool to primary school [6-8], from primary to high school[9-12], and from high school to college or university[13, 14]. Yet, the last opportunity to intervene before adulthood is the transition into post-secondary studies. Therefore, this period is ultimately the most important for establishing lifelong actions such as personal, psychosocial, and movement behaviours for those who have not yet established them[15-17]. Physical activity tends to significantly decrease when graduating from high school and enrolling in universities[18] and among first-year university students[15, 19], with 80% of university students not meeting physical activity recommendations during this transition[14]. These studies show that promoting physical activity opportunities for university students is essential.

Providing university courses that improve student's physical activity levels may be a viable option, as universities can provide students with access to a range of sports facilities, highly educated facilitators, and appropriate technologies[20]. However, establishing and maintaining quality university courses at the university level has been a concern. As such, a guideline developed in the US and China[21-23] suggests that administration/support, assessment, instructional strategies, professionalism, learning environment, program staffing, and curriculum are essential facets that promote quality. Previous studies also suggest that providing strategies for administration and directors[24, 25], modelling the development and support for course instructors[26], and utilising technology[27-29] can be viable strategies for increasing the quality of university courses' that aim to improve the physical activity levels of university students.

There are various factors influence physical activity behaviour in young adults[30]. Gaining knowledge of physical activity is considered one of the principal determining factors of physical activity in university-age students[31]. Study involving 258 adults in Hong Kong found that physical activity knowledge had a positive correlation with levels of physical activity, with this correlation strongest among the university student participants[32]. This finding is also supported by a Chinese cross-sectional study recruiting 9826 university students[33]. This study found that knowledge of physical activity guidelines was correlated with higher physical activity levels[33]. Thus, physical activity knowledge should be considered as one of the learning outcomes of physical education courses in a university setting.

Another important factor that is widely known to be associated with physical activity is motivation [34, 35]. Exercise motivation plays an important role in long-term physical activity behaviour[36]. Systematic reviews examine relationships between motivation and physical activity[37] and examine the effects of physical activity interventions underpinned by motivational principles[38] show that motivation significantly increases physical activity levels[37, 38].

Furthermore, a cross-sectional study involving 1079 participants age 24±9 years showed that motivation for physical activity and exercise is associated with frequency, intensity, and duration of exercise[39]. This finding is also supported by an observational study using a web-based survey involving 320 wearable activity monitor users[40]. This study found that motivational regulation was correlated with moderate to vigorous physical activity[40]. Hence, having motivational outcomes as part of a physical education course in university settings should also be considered.

Research focusing on technologies that promote university students' physical activity levels is gaining more attention. The most common strategy utilised in previous studies has been internet websites41-49. Most reported studies utilising internet websites have been successful in increasing physical activity levels43-49. Another common form of technology utilised to enhance university students' physical activity levels are wearable devices. These have ranged from pedometers50-53 to activity trackers (Misfit, Jawbone UP, Polar M400, Fitbit, and MyWellness Key)54-58. Two of four (50%) studies that utilised a pedometer successfully increased participants mean steps per day53, 59. Two of five (40%) studies using activity trackers also increased student's physical activity levels55, 58. Social media, smartphone applications or mobile apps have also become a technology form that has been used to enhance the physical activity levels of university students60-66. Three of seven (43%) studies using this technology have also successfully increased students' physical activity levels62, 63, 65. However, it is important to highlight that most of the studies utilising technology that aim to increase student's physical activity levels in university settings were non-course-based interventions.

The randomised controlled trial (RCT) aims to test and evaluate the modified prototype with a larger sample size. The prototype of technology-supported physical education includes lecturer website-based learning management system integrated with student's mobile app. This prototype is specifically design and build to support physical education course learning process at university setting aims to increase university students' physical activity levels.

4. AIMS / OBJECTIVES / HYPOTHESES

Aims and Objectives: A prototype LMS, incorporated within a mobile phone app, was specifically designed and built to support a physical education course's learning processes at an Indonesian university. The app aims to increase university students' physical activity levels, knowledge, and motivation. The prototype was tested and modified in 2021 with small sample size. The randomised controlled trial (RCT) will recruit a larger sample size with the aim to test and evaluate the modified prototype Learning Management System (LMS) and mobile app. Students in the intervention group will have access to the modified prototype mobile app that incorporates the LMS. This will enable them to access course content, feedback via their smartphone. Students in the control group will receive the same content using standard teaching methods.

Hypothesis: A technology-supported physical education course intervention will increase university student's physical activity levels, knowledge and motivation more effectively than a non-technology-supported physical education courses..

5. PARTICIPATING SITES

This study is being conducted in one state university (Universitas Pendidikan Indonesia) in Bandung, Indonesia. The physical education course is offered to first-year or second-year

students as a requirement or as an elective course. This university has seven faculties: 1) Faculty of Education; 2) Faculty of Science and Math Education; 3) Faculty of Social Science Education; 4) Faculty of Language, and Literature Education; 5) Faculty of Technology and Vocational Education; 6) Faculty of Sport and Health Education; and 7) Faculty of Arts and Design Education. Students from the Faculty of Sport and Health Education are not required to take a physical education course, while students from the Faculty of Art and Design are required to enrol in the physical education course. For students from the other five faculties, they are allowed to choose between the physical education course or a variety of Arts courses. This organisation will divide students into three groups which will be appropriate for this study: 1) students who are required to enrol in the physical education course; 2) students who choose to enrol in the physical education course; and 3) students who do not choose to enrol and participate in a physical education course.

6. RESEARCH PLAN / STUDY DESIGN

6.1 Type of study

This is a two-arm parallel, randomised controlled trial of a technology-supported physical education course intervention for students enrolled in an elective unit of study. The six lecturers who agree to participate will be randomly assigned to the intervention or control group. Students who enrol randomly by the University system with the participated lecturers of physical education course will be invited to participate in the study. The intervention lecturers will have lecturer training and implement the 16-week technology-supported physical education course intervention for one semester. The Primary outcome is to examine the difference in students' physical activity levels, knowledge and motivation at post-test (at the completion of a 16-week semester) compared with the control group. The secondary outcome is students' experience and enjoyment of the technology-supported physical education course intervention.

6.2 Sample size including power calculation

The sample size calculation is based on the difference in change in the primary outcome (physical activity) from pre- to post-intervention in both group (intervention and control group). Based on previous studies on technology-based physical activity interventions in university students, mean effect sizes of around d = 0.5 are expected in analyses[47, 67]. To detect such intervention effects in two-sided significance testing (α = .05) with a power of 80%, a sample size of 128 participants is required. Taking into account an expected study drop-out of about 20%, 150 participants must be included in the study and participants response rate of about 50%, hence, 300 students (six classes of physical education course) will be invited.

Number of Participants
N=6 physical education course lecturers
(a) n=3 will be assigned to the intervention group,
(b) n=3 will be assigned to the control group.
n=300 students will be randomised 1:1 to either an intervention arm
(a) n=150 a technology-supported physical education course (intervention group)
(b) n=150 non-technology-supported physical education course (control group).

6.3 Statistical analyses

All variables will be checked for normality using the Shapiro-Wilk test. Independent samples t-tests or Mann–Whitney U-tests will then be conducted, as appropriate, to examine: (1) pre-test differences between groups and (2) pre-test differences between students who completed the study and those lost to follow-up. If variables significantly differ between groups, they will be appropriately adjusted in the main analyses. Descriptive statistics will be presented (mean and standard deviation) for each group separately.

Linear mixed models will be used to analyse the differential change between groups on all outcomes from pre-test to post-test, using pre-test data as the covariate. Linear mixed models will be used because these models are robust enough to withstand the biases from missing data. All analyses will be conducted using SPSS 28.0 (Chicago, IL, USA). The level of significance will be set at $p < 0.05$.

6.4 Recruitment and selection of participants*

The randomised controlled trial will include six lecturers and six classes (20 to 50 students each class or n=300). There are two recruitment processes in this RCT including the lecturer and students. The lecturer will be included in the RCT if he/she meets the following inclusion criteria: 1) run two classes of the physical education course; 2) willing to take part in lecturer training before the physical education course class begins; and 3) willing to invite their students to take part in the pilot study. The chief investigator A/Prof Wayne Cotton will be sending the recruitment to applicable members of staff at Universitas Pendidikan Indonesia especially lecturers who run the elective physical education course.

The chief investigator A/Prof Wayne Cotton will be sending the recruitment letter to students enrolled in a physical education course via the participating lecturers (and consenting). Participation in this RCT is voluntary, and all participants will be given Participant Information Statements and invited to sign a Participant Consent Form to participate. Participation or non-participation in this study will not affect students' scores in physical education courses. The number of students in each class varies from 20-50 students. Students will be included in the RCT if they meet the following inclusion criteria: 1) students enrolled in a physical education course with a lecturer who will participate in the pilot study; 2) voluntarily participate in the RCT; and 3) owns an android smartphone. The six lecturers who agree to participate will be randomly assigned to intervention or control. Participation in this RCT is voluntary, and all lecturers and students will be given Participant Information Statements and be invited to sign a Participant Consent Form to participate.

6.5 Informed consent process

The researcher is a current academic staff member at Universitas Pendidikan Indonesia where the study will be conducted, although he is currently on study leave while he is conducting the study. Participation in this study is voluntary. As per the approved pilot study (Project No.: 2021/071), to recruit the course lecturers, the researcher will ask the course coordinator to send an email to course lecturers inviting them to participate in the study. Again, following the approved recruitment processes for the pilot study, the researcher will also ask the consenting course lecturers to invite students who are enrolled in the physical education course to participate in the study. The email will attach the RCT participant information statement and the link to the online RCT consent form. The students who consent to participate in the study will be asked to sign the online RCT consent form. The consent form and the participant information statement will provide the appropriate contact details for those interested in participating. The course lecturers and students who do not consent will not be disadvantaged in any way. Their marks/grades will not be affected nor will their relationship

with the researcher or Universities be affected. If only a small proportion of students agree to participate in the study (i.e., less than 30), the course lecturer will invite students from another physical education class to participate in the study.

Participants will provide written consent to be involved in the RCT (via a RCT consent form). One of the data collection methods is participation in a focus group discussion where they will be asked to provide to oral consent before participating.

6.6 Intervention

The six lecturers who agree to participate will be randomly assigned to intervention or control and will be asked to invite the students who enrol in their physical education course. The intervention lecturer will have lecturer training and implement the technology-supported physical education course for one semester (16 weeks). The outcome will be assessed at pre-test (1st week of physical education course after students agree to participate) and post-test (last week of the physical education course). Students will also be invited to participate in a focus group about their experience using the technology at the end of the course. The intervention group will receive 16 weeks of the physical education course incorporated with a modified prototype.

The type of technology that will be utilised in the RCT is a lecturer site and a student's mobile app that incorporated the physical education course at a university setting. The lecturers' website is entitled PESSPA (Physical Education Supporting Site for Physical Activity), while the student's app is entitled PESAPA (Physical Education Supporting App for Physical Activity). The PESSPA includes the following features (1) Learning management system features such as uploading course materials (text, pdf, picture, video, direct link), creating quizzes, creating assignments, creating discussions for every lesson week (2). Reviewing students goal settings, physical fitness test results, dietary and physical activity records; and (3). Uploading exercise examples and exercise video guidelines. The PESAPA includes the following features: (1) Access course materials (informative texts, pdfs, pictures, videos, etc.), quizzes, assignments submission, and weekly discussions; (2) goal settings opportunities, physical fitness testing protocols, dietary and physical activity recording opportunities; and (3). Access exercise examples and exercise video guidelines.

Figure 1 shows the screenshot of the PESSPA where lecturers are able to manage their physical education course such as adding text, adding file resources, embedding file from google drive and embedding video from youtube. Course material uploaded by lecturers can be access by students via PESAPA (see Figure 2). These figures only show one of the PESSPA and PESAPA feature interpreted the initial design principle one.

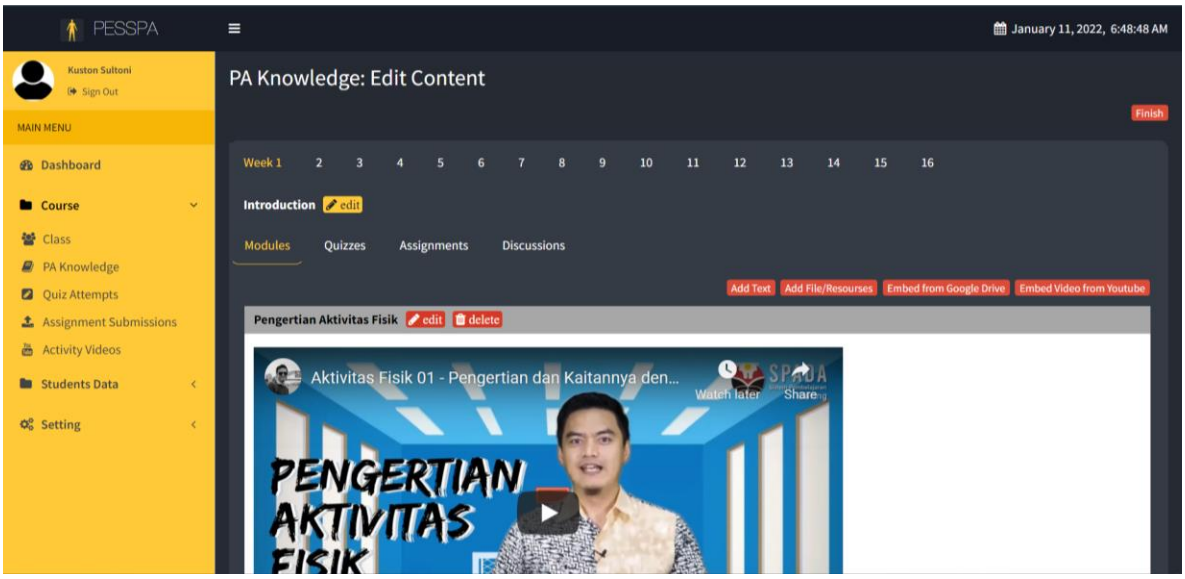

Figure 1. Screenshot of learning management system features on PESSPA.

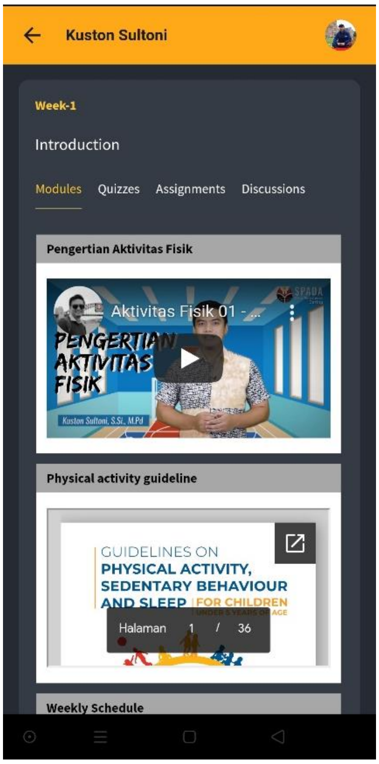

Figure 2. Screenshot of learning management system features on PESAPA.

Table 1. Course content for intervention and control group

Week	Intervention Group	Control Group
1	Course Introduction	Course Introduction
2	Physical activity, Healthy and active lifestyle knowledge	Physical activity, Healthy and active lifestyle knowledge
3	Health-related physical fitness knowledge	Health-related physical fitness knowledge
4	Goal setting in Physical activity and fitness	Goal setting in Physical activity and fitness
5	Body composition	Body composition
6	Aerobic fitness	Aerobic fitness
7	Muscular fitness and flexibility	Muscular fitness and flexibility
8	Midsemester exam	Midsemester exam
9	Nutrition: Healthy eating	Nutrition: Healthy eating
10	Aerobic activity- aerobic dance	Aerobic activity- aerobic dance
11	Aerobic activity- GPS-Based activity	Aerobic activity- GPS-Based activity
12	Muscular strengthening	Muscular strengthening
13	Flexibility training	Flexibility training
14	Create an individual fitness training	Create an individual fitness training
15	Overcome physical activity barrier	Overcome physical activity barrier
16	Final exam	Final exam

7. ETHICAL CONSIDERATIONS

This project is lawful as it is to be conducted under stringent guidelines as set by The University of Sydney Ethic Office Committees. All research conducted will be done so with the approval and consent from all participants.

The research will be conducted in an environment well known to all participants to make them most comfortable. The researcher will ensure to maintain the highest research standards as imposed by the NHMRC. In ensuring to maintain high standards of wellbeing for each participant, open communication lines between the researcher and the participants will be maintained. At no time will participants be pressured to participate in the investigation and may withdraw at any time.

The participants will always have access to the contact details of the researcher and most importantly after the investigation. All participants will be made aware that any questions they have will be answered without judgement and reasonable action will be taken to ensure that they are comfortable with the research taken. If at any time they require further assistance that the researchers cannot provide, the university has a knowledgeable course coordinator whom they and the researchers can work with to provide the necessary assistance the participant requires.

The importance of this research is to develop technology-based physical education courses for university settings so that the results of this study are useful for universities to assist teachers in maintaining and improving physical education courses accompanied by affordable technology for students.

The researchers will endeavour to understand any cultural sensitivities through consultation with the curriculum development team, course coordinator and the Dean of Faculty of Sport and Health Education and from Kuston Sultoni's experiences as academic staff member. Furthermore, the researcher will continue to develop their understanding of culturally sensitive matters through focus group discussion.

As the researcher is a citizen of Indonesia and is current staff member of Universitas Pendidikan Indonesia. He is aware of a large proportion of the issues which exist in a university setting, particularly as a Lecturer of physical education at Universitas Pendidikan Indonesia. Also, he is well aware of the cultural norms, but also the local cultural norms as an academic staff member of Universitas Pendidikan Indonesia.

The USyd student researcher is a current academic staff member at Universitas Pendidikan Indonesia (UPI), however, during the data collection process he will be on study leave from UPI. Therefore, he will not be coordinating or teaching the unit during this time. He also does not have any information regarding which students will enrol the unit. He may however know the teaching staff who will volunteer to participate in this study, however, it will be made clear to them that they can withdraw from the study at any time.

8. SAFETY CONSIDERATIONS

The COVID-19 pandemic situation could impact the proposed study. The first implication regards overseas travel, as this study will be conducted in Indonesia. Hence, safety travel protocols will be followed. This study will also involve class interventions and observations which in the current situation could be conducted in the online environment.

9. OUTCOMES

9.1 Primary outcome

Difference in physical activity levels, knowledge and motivation at 16 weeks' time point compared to physical activity levels measures at baseline.

Three outcomes were measured in the pre-and post-intervention. First, The International Physical Activity Questionnaire-Short Form (IPAQ) was used to measure the student's physical activity levels. IPAQ is internationally recognised as a valid and reliable questionnaire for measuring physical activity levels [68, 69]. The IPAQ is publicly available, it is open access, and no permissions are required to use it. The IPAQ is suitable for adults between 15 and 69 years of age and is primarily used for population surveillance of physical activity levels. The IPAQ short form records the activity of four intensity levels: 1) vigorous-intensity activity such as aerobics, 2) moderate-intensity activity such as leisure cycling, 3) walking, and 4) sitting. Then, to measure student's motivation, the Behavioural Regulation Exercise Questionnaire (BREQ-2) was used which has19 questions using a 5-point Likert scale for responses. BREQ-2 is a valid and reliable measure five subscales of physical activity motivation: Amotivation, External Regulation, Introjected Regulation, Identified Regulation, and Intrinsic Regulation within university students population [70]. Lastly, to measure physical activity knowledge, we developed physical activity knowledge quizzes consisting of 20 multiple choices question based-on the learning outcome of the physical education course. To demonstrate construct validity, two physical education experts reviewed the quizzes whether the questions were

aligned with the learning outcomes. Then, test-retest reliability was performed to measure the consistency of the quizzes with 20 university students within two weeks. The coefficient correlation between test and retest was high r=0.833 (P < 0.05).

9.2 Secondary outcome(s)

- Student's experience using technology-supported physical education app

10. DATA MANAGEMENT

- *Source Data*
  Measurement will be conducted via online questionnaire using RedCap. Audio recording for FGD.
- *Data Capture Methods*
  Audio recordings of focus group discussions will take place to ensure that the conversations and responses are captured correctly and can be transcribed verbatim at a later time. The online survey will be hosted in University of Sydney via RedCap. REDCap is a secure web platform for building and managing online databases and surveys
- *Data Storage*
  Electronic files of consent forms, audio recordings, and interview transcripts will be stored on the University-licensed eNotebook (LabArchives) platform during the project. The eNotebook is an electronic notebook that stores data on secure servers within NSW. The eNotebook is named Tech-supported PE for PA at university Project and will be owned by the student researcher, Kuston Sultoni (UniKey: ksul5404)
- *Record Retention*
  The 5 year period complies with relevant legislation from the State Records Authority of NSW. The study is recruiting participants who are adults (aged older than 18 years).
- *Archiving*
- Study materials will will be stored on the University-licensed eNotebook (LabArchives) platform during the project. The eNotebook is an electronic notebook that stores data on secure servers within NSW. The eNotebook is named Tech-supported PE for PA at university Project and will be owned by the student researcher, Kuston Sultoni (UniKey: ksul5404)

11. TIMELINES / MILESTONES

Predictive timeline for this study:

- Lecturer's recruitment first half of August 2022
- Lecturers training second half of August 2022
- Student's recruitment September 2022
- Pre-test September 2022
- Intervention September 2022 to January 2023
- Post-test January 2023
- Data Analysis January to February 2023
- Publication March 2023

12. FINANCIAL

Usyd student's researcher is supported by the Indonesia Endowment Fund for Education Scholarship (LPDP RI) under a doctoral degree scholarship.

13. PUBLICATION POLICY / DISSEMINATION OF RESULTS

Overall result of this project will be disseminated in journal publications and a PhD thesis.

14. REFERENCES

1. Wilson B and Barnett LM. Physical activity interventions to improve the health of children and adolescents in out of home care – A systematic review of the literature. *Children and Youth Services Review* 2020; 110: 104765. DOI: https://doi.org/10.1016/j.childyouth.2020.104765.
2. Oliveira JS, Sherrington C, Zheng ER, et al. Effect of interventions using physical activity trackers on physical activity in people aged 60 years and over: a systematic review and meta-analysis. *British journal of sports medicine* 2019: bjsports-2018-100324.
3. Hnatiuk J, Brown H, Downing K, et al. Interventions to increase physical activity in children 0–5 years old: a systematic review, meta-analysis and realist synthesis. *Obesity reviews* 2019; 20: 75-87.
4. Kwan RYC, Salihu D, Lee PH, et al. The effect of e-health interventions promoting physical activity in older people: a systematic review and meta-analysis. *European Review of Aging and Physical Activity* 2020; 17: 1-17.
5. Gropper H, John JM, Sudeck G, et al. The impact of life events and transitions on physical activity: A scoping review. *PLOS ONE* 2020; 15: e0234794. DOI: 10.1371/journal.pone.0234794.
6. Oja L and Jürimäe T. Tracking of motor abilities, physical activity, and elementary motor skills during transition from pre-school to school. *Acta Kinesiol Univ Tartuensis* 2001; 6: 91-101.
7. Sigmund E, Sigmundová D and Ansari WE. Changes in physical activity in pre-schoolers and first-grade children: longitudinal study in the Czech Republic. *Child: care, health and development* 2009; 35: 376-382.
8. Jáuregui A, Villalpando S, Rangel-Baltazar E, et al. The physical activity level of Mexican children decreases upon entry to elementary school. *salud pública de méxico* 2011; 53: 228-236.
9. Garcia AW, Pender NJ, Antonakos CL, et al. Changes in physical activity beliefs and behaviors of boys and girls across the transition to junior high school. *Journal of adolescent health* 1998; 22: 394-402.
10. Chong KH, Parrish A-M, Cliff DP, et al. Changes in physical activity, sedentary behaviour and sleep across the transition from primary to secondary school: A systematic review. *Journal of Science and Medicine in Sport* 2020; 23: 498-505.
11. Feu S and de la Cruz Sánchez E. Rural-urban differences in physical activity levels during the transition from primary education to high school. *Revista Espanola de Salud Publica* 2020; 94.
12. Shull ER, Dowda M, Saunders RP, et al. Sport participation, physical activity and sedentary behavior in the transition from middle school to high school. *Journal of Science and Medicine in Sport* 2020; 23: 385-389.
13. Wengreen HJ and Moncur C. Change in diet, physical activity, and body weight among young-adults during the transition from high school to college. *Nutrition journal* 2009; 8: 32.
14. Owens CS, Crone D, De Ste Croix MBA, et al. Physical activity and screen time in adolescents transitioning out of compulsory education: a prospective longitudinal study. *Journal of Public Health* 2013; 36: 599-607. DOI: 10.1093/pubmed/fdt123.

15. Bray SR and Born HA. Transition to university and vigorous physical activity: implications for health and psychological well-being. *J Am Coll Health* 2004; 52: 181-188. 2004/03/17. DOI: 10.3200/JACH.52.4.181-188.
16. Hussey T and Smith P. Transitions in higher education. *Innovations in Education and Teaching International* 2010; 47: 155-164. DOI: 10.1080/14703291003718893.
17. Terenzini PT, Rendon LI, Upcraft ML, et al. THE TRANSITION TO COLLEGE: Diverse Students, Diverse Stories. *Research in Higher Education* 1994; 35.
18. Curry J, Jenkins JM and Weatherford J. Focus on Freshman: Basic Instruction Programs Enhancing Physical Activity. *The Physical Educator* 2015; 72. DOI: 10.18666/tpe-2015-v72-i4-6472.
19. Kimball J, Jenkins J and Wallhead T. Influence of high school physical education on university students' physical activity. *European Physical Education Review* 2009; 15: 249-267.
20. Plotnikoff RC, Costigan SA, Williams RL, et al. Effectiveness of interventions targeting physical activity, nutrition and healthy weight for university and college students: a systematic review and meta-analysis. *Int J Behav Nutr Phys Act* 2015; 12: 45. 2015/04/19. DOI: 10.1186/s12966-015-0203-7.
21. National Association for Sport Physical Education N. Appropriate instructional practice guidelines for higher education physical activity programs. Author. Reston, VA, 2009.
22. Stapleton D and Bulger SM. Adherence to appropriate instructional practice guidelines in US colleges' and universities' physical activity programs. *Journal of Physical Education and Sport Management* 2015; 6: 47-59.
23. Li J. Discussion on Chinese Ordinary University Sports Teaching Pattern in the New Period. *Cross-Cultural Communication* 2012; 8: 75-78.
24. Brock SJ, Russell JA, Cosgrove B, et al. Administrative Strategies for Delivering High-Quality Instruction in a University-Based Physical Activity and Wellness Program. *Kinesiology Review* 2018; 7: 345-349. DOI: 10.1123/kr.2018-0040.
25. Melton BF, Sampson Moore C and Hoffman B. Strategies for College and University Instructional Physical Activity Programs (IPAP) Directors. *International Journal of Higher Education* 2016; 5. DOI: 10.5430/ijhe.v5n1p292.
26. Russell JA. Graduate Teaching-Assistant Development in College and University Instructional Physical Activity Programs. *Journal of Physical Education, Recreation & Dance* 2011; 82: 22-32. DOI: 10.1080/07303084.2011.10598610.
27. Bice MR, Ball JW, Hollman A, et al. Health Technology Use: Implications for Physical Activity Behaviors Among College Students. *International Journal of Kinesiology in Higher Education* 2019; 3: 23-34. DOI: 10.1080/24711616.2018.1516524.
28. Cox DG, Krause JM and Smith MA. Technology in University Physical Activity Courses: A Mini-Ethnographic Case Study. *The Qualitative Report* 2019; 24: 2554-2574.
29. Melton BF and Burdette T. Utilizing Technology to Improve the Administration of Instructional Physical Activity Programs in Higher Education. *Journal of Physical Education, Recreation & Dance* 2011; 82: 27-32. DOI: 10.1080/07303084.2011.10598611.
30. Nahas MV, Goldfine B and Collins MA. Determinants of physical activity in adolescents and young adults: The basis for high school and college physical education to promote active lifestyles. *Physical Educator* 2003; 60: 42.
31. Sallis JF and Owen N. *Physical activity & behavioral medicine*. Thousand Oaks, Calif. ;: Sage, 1999.
32. Hui SS-C, Hui GP-S and Xie YJ. Association between physical activity knowledge and levels of physical activity in chinese adults with type 2 diabetes. *PloS one* 2014; 9: e115098.
33. Abula K, Gröpel P, Chen K, et al. Does knowledge of physical activity recommendations increase physical activity among Chinese college students? Empirical investigations based on the transtheoretical model. *Journal of Sport and Health Science* 2018; 7: 77-82.

34. Bauman AE, Reis RS, Sallis JF, et al. Correlates of physical activity: why are some people physically active and others not? *The lancet* 2012; 380: 258-271.
35. Ryan RM and Deci EL. Self-determination theory and the facilitation of intrinsic motivation, social development, and well-being. *American psychologist* 2000; 55: 68.
36. Richard M, Christina MF, Deborah LS, et al. Intrinsic motivation and exercise adherence. *Int J Sport Psychol* 1997; 28: 335-354.
37. Teixeira PJ, Carraça EV, Markland D, et al. Exercise, physical activity, and self-determination theory: a systematic review. *The international journal of behavioral nutrition and physical activity* 2012; 9: 78-78. DOI: 10.1186/1479-5868-9-78.
38. Knittle K, Nurmi J, Crutzen R, et al. How can interventions increase motivation for physical activity? A systematic review and meta-analysis. *Health psychology review* 2018; 12: 211-230. DOI: 10.1080/17437199.2018.1435299.
39. Duncan LR, Hall CR, Wilson PM, et al. Exercise motivation: a cross-sectional analysis examining its relationships with frequency, intensity, and duration of exercise. *International Journal of Behavioral Nutrition and Physical Activity* 2010; 7: 7.
40. Friel CP and Garber CE. An Examination of the Relationship Between Motivation, Physical Activity, and Wearable Activity Monitor Use. *Journal of Sport and Exercise Psychology* 2020; 1: 1-8.
41. Hastie PA and Sinelnikov OA. The use of web-based portfolios in college physical education activity courses. *Physical Educator* 2007; 64: 21.
42. Skar S, Sniehotta FF, Molloy GJ, et al. Do brief online planning interventions increase physical activity amongst university students? A randomised controlled trial. *Psychol Health* 2011; 26: 399-417. 2010/09/11. DOI: 10.1080/08870440903456877.
43. Magoc D, Tomaka J and Bridges-Arzaga A. Using the web to increase physical activity in college students. *Am J Health Behav* 2011; 35: 142-154. 2011/01/06. DOI: 10.5993/ajhb.35.2.2.
44. Greene, White AA, Hoerr SL, et al. Impact of an online healthful eating and physical activity program for college students. *Am J Health Promot* 2012; 27: e47-58. 2012/11/02. DOI: 10.4278/ajhp.110606-QUAN-239.
45. Hager R, George JD, LeCheminant JD, et al. Evaluation of a university general education health and wellness course delivered by lecture or online. *American Journal of Health Promotion* 2012; 26: 263-269.
46. Everhart K and Dimon C. The impact of course delivery format on wellness patterns of university students. *Education* 2013; 133: 310-318.
47. Sriramatr S, Berry TR and Spence JC. An Internet-based intervention for promoting and maintaining physical activity: a randomized controlled trial. *American journal of health behavior* 2014; 38: 430-439.
48. Kattelmann KK, White AA, Greene, et al. Development of Young Adults Eating and Active for Health (YEAH) internet-based intervention via a community-based participatory research model. *J Nutr Educ Behav* 2014; 46: S10-25. 2014/01/25. DOI: 10.1016/j.jneb.2013.11.006.
49. Duan YP, Wienert J, Hu C, et al. Web-based intervention for physical activity and fruit and vegetable intake among Chinese university students: a randomized controlled trial. *Journal of medical Internet research* 2017; 19: e106.
50. Perkins J. *Baseline comparison and the effects of education and pedometer use on physical activity in undergraduate students in psychology and personal health courses*. Central Michigan University, Ann Arbor, 2006.
51. LeCheminant JD, Smith JD, Covington NK, et al. Pedometer use in university freshmen: a randomized controlled pilot study. *Am J Health Behav* 2011; 35: 777-784. 2012/01/19. DOI: 10.5993/ajhb.35.6.13.
52. Sharp P and Caperchione C. The effects of a pedometer-based intervention on first-year university students: A randomized control trial. *J Am Coll Health* 2016; 64: 630-638. 2016/10/30. DOI: 10.1080/07448481.2016.1217538.
53. Miragall M, Dominguez-Rodriguez A, Navarro J, et al. Increasing physical activity through an Internet-based motivational intervention supported by pedometers in a

sample of sedentary students: A randomised controlled trial. *Psychol Health* 2018; 33: 465-482. 2017/09/08. DOI: 10.1080/08870446.2017.1368511.
54. Melton BF, Buman MP, Vogel RL, et al. Wearable devices to improve physical activity and sleep: a randomized controlled trial of college-aged African American women. *Journal of Black Studies* 2016; 47: 610-625.
55. Rote AE. Physical activity intervention using Fitbits in an introductory college health course. *Health Education Journal* 2017; 76: 337-348. DOI: 10.1177/0017896916674505.
56. Maselli M, Gobbi E and Carraro A. Effectiveness of individual counseling and activity monitors to promote physical activity among university students. *The Journal of sports medicine and physical fitness* 2017; 59: 132-140.
57. Kim Y, Lumpkin A, Lochbaum M, et al. Promoting physical activity using a wearable activity tracker in college students: A cluster randomized controlled trial. *J Sports Sci* 2018; 36: 1889-1896. 2018/01/11. DOI: 10.1080/02640414.2018.1423886.
58. Pope ZC, Barr-Anderson DJ, Lewis BA, et al. Use of Wearable Technology and Social Media to Improve Physical Activity and Dietary Behaviors among College Students: A 12-Week Randomized Pilot Study. *International Journal of Environmental Research and Public Health* 2019; 16: 3579.
59. Ornes L and Ransdell LB. Web-based physical activity intervention for college-aged women. *International Electronic Journal of Health Education* 2007; 10: 126-137.
60. Melton BF, Bland H, Harris B, et al. Evaluating a Physical Activity App in the Classroom: A Mixed Methodological Approach Among University Students. *The Physical Educator* 2015; 72. DOI: 10.18666/tpe-2015-v72-i4-7139.
61. Bruening M, Van Woerden I, Todd M, et al. A mobile ecological momentary assessment tool (devilSPARC) for nutrition and physical activity behaviors in college students: a validation study. *Journal of medical Internet research* 2016; 18: e209.
62. Tong HL, Coiera E, Tong W, et al. Efficacy of a Mobile Social Networking Intervention in Promoting Physical Activity: Quasi-Experimental Study. *JMIR Mhealth Uhealth* 2019; 7: e12181. 2019/03/29. DOI: 10.2196/12181.
63. Pentakota N, Ramaswamy G, Thekkur P, et al. Is a smartphone application effective in improving physical activity among medical school students? Results from a quasi-experimental study. *Int J Adolesc Med Health* 2019: 1-8. 2019/03/29. DOI: 10.1515/ijamh-2018-0192.
64. Edney SM, Olds TS, Ryan JC, et al. A Social Networking and Gamified App to Increase Physical Activity: Cluster RCT. *Am J Prev Med* 2020; 58: e51-e62. 2020/01/22. DOI: 10.1016/j.amepre.2019.09.009.
65. Wang M, Guo Y, Zhang Y, et al. Promoting healthy lifestyle in Chinese college students: evaluation of a social media-based intervention applying the RE-AIM framework. *European journal of clinical nutrition* 2020; 75: 335-344. DOI: 10.1038/s41430-020-0643-2.
66. Krzyzanowski MC, Kizakevich PN, Duren-Winfield V, et al. Rams Have Heart, a Mobile App Tracking Activity and Fruit and Vegetable Consumption to Support the Cardiovascular Health of College Students: Development and Usability Study. *JMIR mHealth and uHealth* 2020; 8: e15156.
67. Okazaki K, Okano S, Haga S, et al. Development and its Evaluation of the Distance and Interactive Proper System for College Students that Promotes Physical Activity through the Internet. *Educational technology research* 2010; 33: 85-93.
68. Craig C, Marshall A, Sjostrom M, et al. International Physical Activity Questionnaire-Short Form. *J Am Coll Health* 2017; 65: 492-501.
69. Craig CL, Marshall AL, Sjöström M, et al. International physical activity questionnaire: 12-country reliability and validity. *Medicine & science in sports & exercise* 2003; 35: 1381-1395.
70. D'Abundo ML, Sidman CL, Milroy J, et al. Construct validity of college students' responses to the behavioral regulation in exercise questionnaire (BREQ-2). *Recreational Sports Journal* 2014; 38: 40-49.
