# Supplementary material for: Using a design-based research approach to develop a technology-supported physical education course to increase the physical activity levels of university students: Study protocol paper
Source: PLoS One. 2022 Dec 1;17(12):e0269759. doi: 10.1371/journal.pone.0269759 (PMC9714829; doi:10.1371/journal.pone.0269759)
Supplement: Attachment — Submitted filename: Rebuttal Letter PLOS one Kuston (Final).pdf [file pone.0269759.s004.pdf]

THE UNIVERSITY OF SYDNEY
ABN 15 211 513 464

Discipline of Health and Physical Education
Sydney School of Education and Social Work
Faculty of Arts and Social Science

Rebuttal letter

Kuston Sultoni[1,2*], Louisa Peralta[1] and Wayne Cotton[1]
[1]Sydney School of Education and Social Work, The University of Sydney,
[2]Faculty of Sports and Health Education, Universitas Pendidikan Indonesia,
*ksul5404@uni.sydney.edu.au

August 18, 2022

Dear Editorial Board of PLOS ONE,

We would like to thank the editor and reviewers for the opportunity to revise and resubmit our manuscript PONE-D-22-15083 entitled "Using a design-based research approach to develop a technology-supported physical education course to increase the physical activity levels of university students: Study protocol paper."

We found the reviewers' comments helpful and as a result we carefully considered and responded to each suggestion. In all cases, we were successful in incorporating the reviewers' feedback and have revised the manuscript accordingly. We have corrected minor errors as well as made appropriate formatting changes throughout the manuscript as follow:

1. Please ensure that your manuscript meets PLOS ONE's style requirements, including those for file naming. The PLOS ONE style templates can be found at https://journals.plos.org/plosone/s/file?id=wjVg/PLOSOne_formatting_sample_main_body.pdf and https://journals.plos.org/plosone/s/file?id=ba62/PLOSOne_formatting_sample_title_authors_affiliation.

   Response:
   We have revised the manuscript according to PLOS ONE style requirements.

2. Please expand the acronym "LPDP RI" as indicated in your financial disclosure so that it states the name of your funders in full.
   This information should be included in your cover letter; we will change the online submission form on your behalf.

   Response:
   We have expanded the acronym "LPDP RI" as "Lembaga Pengelola Dana Pendidikan Republik Indonesia" please see Page 13 Line 18.

3. Please include your full ethics statement in the 'Methods' section of your manuscript file. In your statement, please include the full name of the IRB or ethics committee who approved or waived your study, as well as whether or not you obtained informed written or verbal consent. If consent was waived for your study, please include this information in your statement as well.

   Response:
   We have included full ethics statement in the Method section under Study Design subsections Page 4 Line 16-19.

   > *"This project has obtained ethics approval from the University of Sydney Human Research Ethics Committee (Project No. 2021/071; Project No. 2021/935). Written informed consent was obtained from all participants before the study commenced"*

4. We note that the original protocol file you uploaded contains a confidentiality notice indicating that the protocol may not be shared publicly or be published. Please note, however, that the PLOS Editorial Policy requires that

the original protocol be published alongside your manuscript in the event of acceptance. Please note that should your paper be accepted, all content including the protocol will be published under the Creative Commons Attribution (CC BY) 4.0 license, which means that it will be freely available online, and any third party is permitted to access, download, copy, distribute, and use these materials in any way, even commercially, with proper attribution.
Therefore, we ask that you please seek permission from the study sponsor or body imposing the restriction on sharing this document to publish this protocol under CC BY 4.0 if your work is accepted. We kindly ask that you upload a formal statement signed by an institutional representative clarifying whether you will be able to comply with this policy. Additionally, please upload a clean copy of the protocol with the confidentiality notice (and any copyrighted institutional logos or signatures) removed.

Response:
We have removed the confidentiality notice and institutional logos from the original protocol file

5. We note that the original protocol that you have uploaded as a Supporting Information file contains an institutional logo. As this logo is likely copyrighted, we ask that you please remove it from this file and upload an updated version upon resubmission.

Response:
We have removed the institutional logos from the original protocol file

6. Please include captions for your Supporting Information files at the end of your manuscript, and update any in-text citations to match accordingly. Please see our Supporting Information guidelines for more information: http://journals.plos.org/plosone/s/supporting-information.

Response:
We have revised the in-text citations for supporting information (see Page 6 Line 41 and Page 10 Line 13) and included the supporting information files caption at the end of the manuscript (see Page 19 Line 8-11)

7. Please review your reference list to ensure that it is complete and correct. If you have cited papers that have been retracted, please include the rationale for doing so in the manuscript text, or remove these references and replace them with relevant current references. Any changes to the reference list should be mentioned in the rebuttal letter that accompanies your revised manuscript. If you need to cite a retracted article, indicate the article's retracted status in the References list and also include a citation and full reference for the retraction notice.

Response:
We have reviewed the reference list. We added three references to the discussion section.

Additional Editor Comments:

1. The discussion section needs to be rewritten.

Response:
We have rewritten the discussion section.

2. Reviewer #1: The subject of this study is judged to be very interesting. Physical activity is an important activity for all of us.
Approaching the seven research programs in this study is judged to be creative. However, it is judged that the discussion part is somewhat weak. Further discussion on the results of this study is needed. It is judged that the quality of the paper will be improved if the discussion of each research stage is added.
On page 9, please correct the notation of prior research in the form.

Response:
Thank you for your comment. We have rewritten the discussion section (see Page 12 Line 21-29).

> *"The systematic review study of this project has been completed and published [85]. Four design principles generated from the review will be confirmed and enhanced in the series of focus group discussions with key stakeholders (Phase 1) before the prototype is built from the initial design principles (Phase 2). The prototype will be tested and evaluated in the small sample pilot study to ensure prototype feasibility and validity (Phase 3). Then modifications will be performed based on the pilot study findings (Phase 4) with the modified prototype (Phase 5) tested and evaluated in a large*

randomised controlled trial study (Phase 6). The final design principles will then be created (Phase 7) to aid future research and practitioners who will design, implement, and evaluate university physical education courses."

3. Reviewer #2: The endeavor of the authors are appreciated, as the topic seems to be relevant and promising. thanks alot for your protocol topic selection

   Response:
   Thank you for your comment.

Thank you for your consideration of our revised manuscript.

Sincerely,

Kuston Sultoni
