# Supplementary material for: Using a design-based research approach to develop a technology-supported physical education course to increase the physical activity levels of university students: Study protocol paper
Source: PLoS One. 2022 Dec 1;17(12):e0269759. doi: 10.1371/journal.pone.0269759 (PMC9714829; doi:10.1371/journal.pone.0269759)
Supplement: Attachment — Submitted filename: Rebutal Letter PLOS one Kuston (2) 30092022.pdf [file pone.0269759.s005.pdf]

Rebuttal letter

Kuston Sultoni[1,2*], Louisa Peralta[1] and Wayne Cotton[1]
[1]Sydney School of Education and Social Work, The University of Sydney,
[2]Faculty of Sports and Health Education, Universitas Pendidikan Indonesia,
*ksul5404@uni.sydney.edu.au

September 29, 2022

Dear Editorial Board of PLOS ONE,

We would like to thank the editor and reviewers for the opportunity to revise and resubmit our manuscript PONE-D-22-15083 entitled "Using a design-based research approach to develop a technology-supported physical education course to increase the physical activity levels of university students: Study protocol paper."

Please find our responses to the reviewers' comments as follow:

Reviewer #1:

1. This study is considered very interesting. Physical education in particular seems to be a subject that is being studied in all countries.

Response:
Thank you for your comment.

2. However, it is found that the reference in the introduction is incorrect.

Response:
Thank you for your comment. We have double-checked the reference list, we did not find the incorrect reference in the introduction.

3. At the end of the introduction, the word aim and the word purpose are used indiscriminately and redundantly, so they need to be corrected. In addition, the purpose of the study is explained again, and it is judged that the introduction has been sufficiently explained.

Response:
Thank you for your feedback on the last paragraph. We have revised the last paragraph to align with the feedback. It now reads:

> *The aim of this design-based research project is to create a series of design principles, develop an intervention based on the design principles and measure the impact of the intervention [68-70]. The impact of the intervention will be measured through an increase in physical activity levels, knowledge, and motivation. The purpose of this protocol paper is to fully detail the plan for conducting a design-based research approach to develop a technology-supported physical activity course for increasing university students' physical activity knowledge, motivation and levels in a comprehensive, transparent manner, contributing to the methodological evidence base in this field.*

4. The research method was well described.

Response:
Thank you for your comment.

5. No study results are presented. This is a very dangerous the paper.

Response:
As this paper is a study protocol paper, we consider it very low risk as no results are presented. Please check protocol paper guidelines on PlosOne: https://journals.plos.org/plosone/s/journal-information#loc-scope

6. In the discussion, the results of the study were not compared and analyzed with previous studies. This study lacks the interpretation or argument of the researcher to clarify the necessity. This study is considered insufficient for publication in the journal plos.

Response:
As this paper is a study protocol paper, therefore no study results are discussed in the discussion section.

Reviewer #2:

1. The study potocol was well-designed and the statistics are good

   Response:
   Thank you for your comment.

Thank you for your consideration of our manuscript.

Sincerely,

Kuston Sultoni
